# Transepithelial Photorefractive Keratectomy Compared to Conventional Photorefractive Keratectomy: A Meta-Analysis

**DOI:** 10.1155/2022/3022672

**Published:** 2022-08-23

**Authors:** Tariq Alasbali

**Affiliations:** Department of Ophthalmology, College of Medicine, Imam Mohammed Ibn Saud Islamic University, Riyadh, Saudi Arabia

## Abstract

This meta-analysis review compares the primary and secondary outcomes of transepithelial photorefractive keratectomy (TPRK) to the conventional photorefractive keratectomy (PRK), in terms of efficacy, predictability, safety, and patient perspectives. A total of 1711 eyes with PRK (811 eyes) and TPRK (900 eyes) from 12 studies were included through bibliographic searches. The main outcomes were efficacy, predictability, and safety parameters, and the secondary outcomes included visual and patient-reported parameters. The effect measures were weighted mean differences with 95% confidence intervals (CI) which were derived from the random-effects model of the meta-analysis to account for possible heterogeneity. TPRK procedure presents a comparable status in the main outcome and a very dominant significance in all the secondary outcomes in this meta-analysis. This study updates the evidence of the accuracy of TPRK procedure for surgical correction of all refractive errors and was deemed safer with less surgical time required and an early healing time.

## 1. Introduction

The leading cause of visual impairment worldwide is due to uncorrected refractive errors [1]. Laser corneal refractive surgery has emerged as an effective alternative to optical correction of refractive errors with glasses or contact lenses [2]. A wide range of surgical techniques have been developed that change the refractive error of the eye by removing the corneal tissue and reshaping the cornea. The various types of corneal refractive surgery have a range of individual advantages and disadvantages [3].

Surface corneal refractive surgery is an effective and safe choice for some special patients, especially for the corneal epithelial basement membrane lesions, and a thin cornea with high myopia [4]. Development of the excimer laser in 1983 opened the world of refractive surgery, giving rise to procedures such as photorefractive keratectomy (PRK), transepithelial photorefractive keratectomy (TPRK), laser in situ keratomileusis (LASIK), and small-incision lenticule extraction (SMILE) [5].

Photorefractive keratectomy (PRK) employs an excimer laser ablation of the anterior corneal stroma beneath the epithelium [6, 7]. Conventionally, the corneal epithelium is manually scraped before the ablation. Mechanical debridement and epithelial removal time are some of the issues faced in refractive surgeries. Many techniques have been developed and adopted including laser transepithelial debridement, diluted ethanol, and a rotating brush [8].

A single-step laser epithelial removal and stromal ablation is performed in TPRK which removes the epithelial layer in a precise and uniform manner, with minimal chance of unequal stromal hydration [9]. Introduced in September 2009, TPRK has undergone many modifications, like the removal of the corneal epithelium with laser phototherapeutic ablation which provides the desired refraction corrections [10]. Unlike the conventional photorefractive keratectomy (PRK), where manual mechanical scraping or an alcohol solution is not needed in TPRK and there is no contact of any surgical equipment with the cornea [11]. These factors have influenced the outcomes like postoperative pain, lessened epithelial healing time, dry eyes, and a shortened surgical time. Explicit evidence is vital to support the chosen procedure of refractive keratectomy which enhances the patient outcome and comfort.

Both PRK and TPRK ablates the corneal stroma to correct the errors, and hence has a common base to be compared for the patient preference [12]. Comparison of PRK to TPRK has been a subject of many studies. Most of these studies have concentrated in comparing the visual and refractive outcomes with variable results [13–17]. The pain score and patient satisfaction are the outcomes of importance reflecting the societal acceptance of a procedure. Very few studies have highlighted the impact on the importance of patient-reported outcomes of these two techniques [18–20]. Lower postoperative pain was reported with one-step TPRK compared to the conventional PRK with the same visual and refractive primary and secondary outcomes [18, 19]. The association between the surgical outcomes and the patient satisfaction has been explored by only a few studies [8, 21].

The purpose of this study was to compare the primary and secondary visual and refractive outcomes, along with the pain score and patient satisfaction between the conventional PRK with TPRK. It is apparent from the studies of the last two decades that there has been no recent and most updated studies reporting a meta-analysis comparing the refractive and patient-reported outcomes between the conventional PRK and TPRK. This study aims to provide an updated meta-analysis-based evidence by investigating the surgical and patient outcomes of PRK with TPRK and aims to provide clinical guidance in selection of the procedure.

## 2. Methods

This meta-analysis complies with the PRISMA (Preferred Reporting Items for Systematic Reviews and Meta-Analyses) [22]. statement. This study adheres to the all the steps advised in Cochrane Handbook of systematic reviews of intervention [23].

### 2.1. Search Strategy and Data Extraction

A thorough bibliographic search of the electronic databases of PubMed, MEDLINE, EMBASE, and the Cochrane Library was undertaken in this study. Boolean logics were used for the keywords PRK versus TPRK, photorefractive surgical outcomes, alcohol-assisted photorefractive keratectomy, conventional PRK, transepithelial keratectomy, refractive keratectomy, and refractive error surgeries for searching the relevant literature. The studies were independently identified, selected, and appraised. To avoid possible selection bias, grey literature including published and unpublished thesis was obtained by searching the Web of Science, ProQuest Dissertations, and https://clinicaltrials.gov.

The reference sections of the retrieved original articles and reviews were scanned for studies that might have been missed in the primary searches.

Studies were filtered with regard to study design and the methodological features, and the visual, refractive, and the patient-reported outcomes were evaluated under this study.

The participants, intervention, comparisons, outcomes (PICO) of the current meta-analysis were as follows.

Participants (P): all age groups of patients, diagnosed with refractive errors have undergone either the conventional PRK or TPRK. Intervention (I): transepithelial photorefractive keratectomy for surgical refractive error correction. Comparisons (C): conventional photorefractive keratectomy for the surgical refractive error correction. Outcomes (O): postoperative visual and refractive outcomes along with patient-reported outcomes.

The full text articles were assimilated that were relevant after the review of the titles and abstracts. The final eligibility assessment was performed independently for full text articles and all studies were individually appraised.

**Figure 1:**
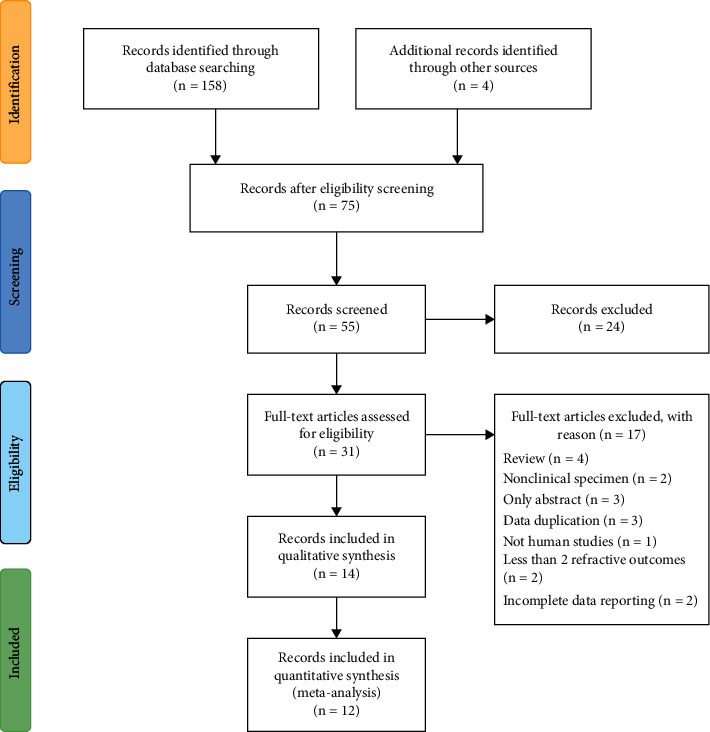
PRISMA flow diagram study selection process.

Inclusion criteria for the studies were as follows: (1) all kinds of experimental and interventional studies from 2011 until 2021, comparing the PRK and TPRK for all types of refractive errors and patient population, (2) studies which included at least 3 of the following postoperative outcomes: uncorrected distance visual acuity (UDVA), postoperative spherical equivalent (SE), postoperative best corrected distance visual acuity (BCDVA), corneal haze, and early postoperative pain score.  Figure 1 illustrates the PRISMA flow diagram for the studies selected in the search process and eligibility appraisal.

(1) Animal studies, (2) reviews, letters, editorials, survey reports, and abstracts were only available, (3) studies with descriptive results and outcomes were not numerically reported, and (4) non-English articles were excluded.

Review manager 5.4.1 (RevMan, Cochrane Collaboration, and Oxford, UK) was used to manage, analyze, and synthesize the included study data. The institutional research board and ethics committee ruled out that approval was not required for this study being a review study.

### 2.2. Data Extraction and Assessment of the Risk of Bias

Data extraction of the included studies was performed using a standard data extraction form in Excel. All relevant information on the included studies was extracted, including participant and intervention characteristics, postoperative main and secondary outcomes, postoperative follow-up period, type of laser used, and industrial funding or influence on the study.

**Figure 2:**
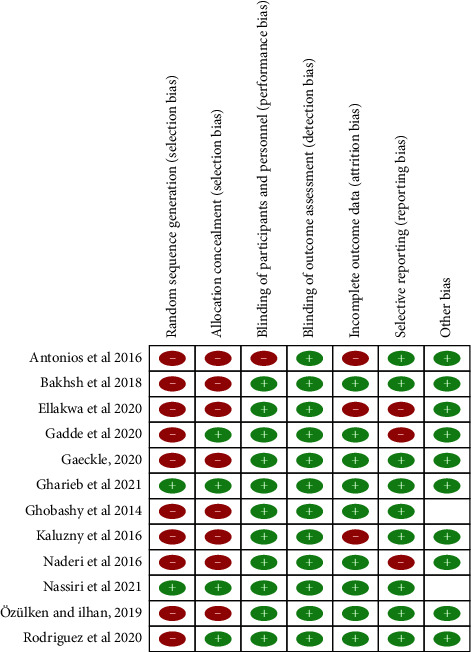
Risk of bias summary about the methodological quality of studies included using the Cochrane risk of bias tool. Symbols show low risk of bias shown as “+”, unclear risk of bias, or other bias shown as a blank space, or high risk of bias shown as “−”.

The risk of bias method from the Cochrane Collaboration was used [23]. to appraise the quality of the included studies. The studies were graded as low, high, or unclear risk of bias for each of the following items using this method. The domains included in this grading of risk of bias were the random sequence generation and allocation concealment (both items relate to selection bias), masking of participants and personnel (detection bias), incomplete outcome data (attrition bias), selective reporting (reporting bias), and other biases.  Figure 2 is descriptive of the risk of bias tool used in this study.

### 2.3. Outcome Measures

The refractive outcomes compared in this study were divided into postoperative primary and secondary outcomes.

### 2.4. Primary Outcomes

The primary outcomes factors were for the efficacy, safety, and predictability of the techniques under comparison.

The data of interest for each clinical outcome were extracted as follows: (1) efficacy: the number of eyes postoperatively achieving an uncorrected distance visual acuity (UDVA) of 20/20 or better, (2) predictability: the number of eyes achieving a postoperative spherical equivalent (SE) within 0.50 diopter (D) of the intended visual outcome, and (3) safety: the number of eyes that lost 2 or more lines of postoperative best corrected distance visual acuity (BCDVA) relative to the preoperative corrected distance visual acuity CDVA [17, 24, 25].

### 2.5. Secondary Outcomes

This meta-analysis went deeper into almost all factors influencing the postoperative outcomes of the techniques under comparison. The factors considered among the secondary outcomes included the following: (1) healing time of the corneal epithelium, (2) corneal haze to rule out postoperative complications, (3) postoperative dry eye symptoms, (4) visual acuity at night, (5) postoperative astigmatism, (6) total surgical time, (7) corneal epithelial healing time, (8) early postoperative pain score and (9) patient satisfaction.

The surgical time is a helpful factor in identifying the variable factors leading to the surgical complications such as postoperative dry eye symptoms [26]. Patient satisfaction was an important factor included as the secondary outcome in this review as it reveals the societal preference to the procedure and readiness to repeat the procedure. As the follow-up time varied from 3 months to 40 months, the data provided at the end of the follow-up period were used for comparison.

### 2.6. Data Synthesis

Methodological differences or heterogeneity among the included studies can potentially affect the pooled results of the study and hence the statistical analysis and the assessment of the heterogeneity was performed for each reported outcomes in the included studies. For the continuous outcomes, the weighted mean difference (WMD) with 95% confidence interval (CI) was calculated. A pooled odds ratio (OR) was calculated for the studies with dichotomous outcomes, if the OR cannot be calculated, the pooled risk ratio (RR) with 95% CI was calculated.

**Figure 3:**
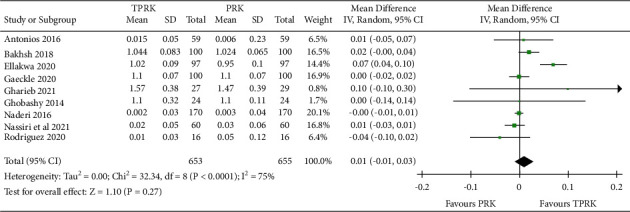
Forest plot of postoperative UDVA comparison between TPRK and the conventional PRK.

Heterogeneity was also assessed, and an *I*^2^ value greater than 50% was considered significant. In this instance, a random-effects model was used because it gives a more conservative estimate and is less influenced by the weighting of each study [27]. A *P* value of less than 0.05 was considered statistically significant. All statistical analyses were performed by the RevMan software (version 5.0, Oxford, United Kingdom). When the level of heterogeneity among the included studies was less than 50%, a fixed-effect model was used. Publication bias was assessed visually with a funnel plot, [28]. as shown in Figure 3. The meta-analysis was performed with the Review manager 5.4.1 (RevMan, Cochrane Collaboration, and Oxford, UK).

## 3. Results

### 3.1. Study Search

A total of 1711 eyes of 957 patients were included in this meta-analysis from the studies published between 2011 and 2021. This included 811 eyes which underwent conventional PRK and 900 eyes which underwent the TPRK procedures. There were 12 articles included in the meta-analysis which were relevant to the search terms. 75 studies were excluded after abstract evaluation (Figure 1).  Table 1 provides the summary of the attributes included in the studies.

### 3.2. Characteristics and Quality of Trials

In relation to the masking of participants and personnel, almost most of the trials were rated at “high risk of bias” (10 of 12 trials, 83.33%); as for the attrition bias and reporting bias, the majority of trials were rated at “low risk of bias” because they reported the complete outcome data (11 out of 12 trials, 91.66%) and did not selectively report outcomes (1 out of 12 trials, 8.33%). There were studies at “unclear risk of bias” with issues relating to random sequence generation, allocation concealment, and masking of outcome assessment.

(0 out of 12 trials, 0%).  Figure 2 shows the risk of bias summary based on the review quality appraisal judgements about each risk of bias item for each included study.

The included studies were each from Colombia [36], Lebanon [32], the Kingdom of Saudi Arabia [33], India [34], and Turkey [11]. There were 2 studies from Iran [29, 30], 2 from Germany [31, 35], and 3 studies from Egypt [29, 37, 38]. Eight studies were prospective and 3 were retrospective studies. Two studies [29, 38]. were randomized clinical trials (RCTs). The patient selection included different degrees of myopia with or without astigmatism, with no other ophthalmologic pathology. The length of postoperative follow-up varied from 3.5 to 40 months.  Table 1 contains the characteristics of the studies [11, 29–38]. Five studies [29, 30, 35, 37]. report that TPRK has a lead in postoperative outcomes like the predictability, epithelial healing, pain score, and patient satisfaction. Early postoperative outcomes were reported to be significantly better [33]. with TPK by one study while stating the similarity in both procedures after 6 months of follow-ups. Five studies [11, 31, 32, 34, 37, 38]. report the equivalent comparability between TPRK and PRK in all the visual and refractive postoperative outcomes.

As there were multiple studies observing the same effect overlapped with one another, the forest plot was used to present the results. If the results were very close or similar between the underlying studies, the data were said to be homogeneous, and the tendency for these data was to be more conclusive. The heterogeneity was indicated by the *I*^2^. Heterogeneity of less than 25% was termed low and indicated a greater degree of similarity between the study data, and the *I*^2^ value of more than 50% indicated more dissimilarity [39].

### 3.3. Main Outcome Comparison

**Figure 4:**
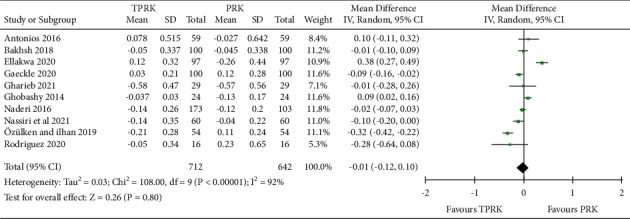
Comparison of the forest plot of the postoperative SE between TPRK and the conventional PRK.

The forest plot in Figure 4 is illustrative of the primary postoperative outcomes comparison results of the two techniques under this study.

#### 3.3.1. Efficacy: Uncorrected Distance Visual Acuity (UDVA)

Meta-analysis in relation to efficacy was performed for 9 studies [29, 30, 32, 33, 35, 37, 38]. with continuous outcomes from the included 12 studies. The efficacy of the forest plot showed a statistically favorable stance for TPRK for the efficacy factor in comparison to the conventional PRK. Figure 3 is illustrative of the pooled effect of all primary outcomes considered under this study. These included studies had varied follow-up periods which affected the pooled effect results. The studies with the longer period of follow-up contributed to the assumption of no significant difference between the two procedures when the postoperative UDVA was compared.

The assimilated pooled studies showed a statistically comparable result in the UDVA of 20/20 in the eyes who underwent TPRK compared to PRK and had a mean difference (IV, Random, 95% CI) of 0.01 (−0.01, 0.03). Pooled studies show heterogeneity as Tau^2^ = 0.00; Chi^2^ = 32.30, *df* = 8 (*P* < 0.0001); *I*^2^ = 75%. The test for overall effect is *Z* = 1.07 (*P*=0.29).

#### 3.3.2. Predictability: Postoperative Spherical Equivalent SE

This meta-analysis was performed for 10 studies [11, 29, 31–33, 36–38]. from the included studies with continuous outcomes, to compare the predictability nature (postoperative SE) of the two procedures under this study.

The assimilated pooled studies favored TPRK in the predictability which can be translated as the accuracy of the procedure. The result in the postoperative SE in the eyes who underwent TPRK compared to PRK was mean difference (IV, Random, 95% CI) −0.01 (−0.12, 0.10). Pooled studies show heterogeneity: Tau^2^ = 0.00; chi^2^ = 32.33, *df* = 8 (*P* < 0.0001); and *I*^2^ = 75%. The test for the overall effect is *Z* = 1.10 (*P*=0.27). The heterogeneity in the results is explored in the discussions. The forest plot results for this are shown in Figure 4. The forest plot for this comparison showed a significantly better predictability between TPRK and the conventional PRK.

#### 3.3.3. Safety: Best Corrected Distance Visual Acuity (BCDVA)

**Figure 5:**
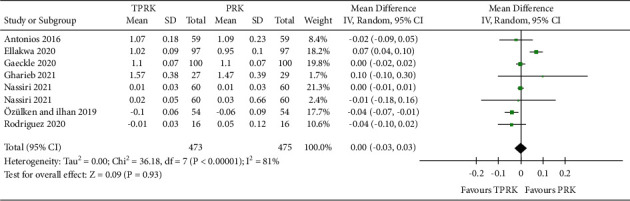
Comparison of the forest plot of postoperative BCDVA between TPRK and the conventional PRK.

Data for this outcome were collected from 6 studies [29, 30, 36]. with continuous outcome reports. The safety comparison between the two procedures were pooled by meta-analysis statistics. The forest plot showed a statistically equivalent treatment of safety between the conventional PRK and TPRK.  Figure 5 is illustrative of the pooled effect of all primary outcomes considered under this study.

The assimilated pooled studies are homogeneous and showed an almost equivalent status in the postoperative BCDVA with the TPRK procedure in comparison to the conventional PRK (Mean Difference (IV, Random, 95% CI), 0.00 [−0.03, 0.03]. Pooled studies show Heterogeneity: Heterogeneity: Tau^2^ = 0.00; chi^2^ = 36.18, *df* = 7 (*P* < 0.0001); and *I*^2^ = 81%. The test for the overall effect is *Z* = 0.09 (*P*=0.93).

### 3.4. Secondary Outcome Comparison

**Figure 6:**
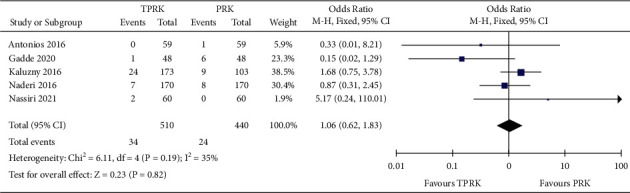
Comparison of the forest plot of postoperative corneal haze dichotomous outcome between TPRK and the conventional PRK.

Only these 9 studies [29–33, 35, 37, 38]. reported the secondary outcomes of concern for this comparison study. Meta-analysis was performed for the secondary outcomes reported from these 9 studies. Five studies [29–32, 34]. reported dichotomous outcomes for corneal haze postoperatively. Forest plot of Figure 6 is illustrative of the postoperative corneal haze comparison between the two procedures in this study. Forest plot showed comparable and equivalent incidence in postcorneal haze with both the procedures and hence both procedures can be inferred to have minimal postoperative complication.

Postoperative incident of corneal haze in both procedures: the pooled data revealed an OR of 0.96 (95% CI, 0.62 to 1.83). Heterogeneity: chi^2^ = 6.11, *df* = 4 (*P*=0.19); *I*^2^ = 35%. Test for overall effect: *Z* = 0.23 (*P*=0.82).

Only 2 studies from the included studies [31, 33]. reported patient satisfaction levels. Both the studies showed that TPRK had a strong dominance in patient satisfaction and acceptance.

Pain score was reported by 7 studies [29–33, 35, 38]. as continuous outcome measures and 1 study [37]. as dichotomous outcome. Figure 6 shows the postoperative secondary outcome comparison between TPRK and the conventional PRK from the continuous outcomes.

**Figure 7:**
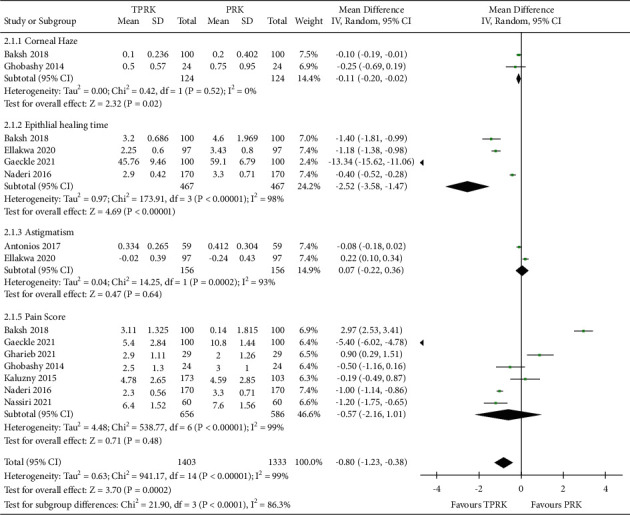
Comparison of the forest plot of the secondary outcomes in the study between TPRK and the conventional PRK.

It can be seen from the aggregate pooled effect from the forest plot of Figure 7 that TPRK has clear and dominant advantage over the conventional PRK in all the secondary postoperative outcomes. Mean Difference (IV, Random, 95% CI), −0.80 (−1.23, −0.38).

Heterogeneity: Tau^2^ = 0.63; Chi^2^ = 941.17, *df* = 14 (*P* < 0.00001); *I*^2^ = 99%. Test for overall effect: *Z* = 3.70 (*P*=0.0002). Test for subgroup differences: Chi^2^ = 21.90, *df* = 3 (*P* < 0.0001), *I*^2^ = 86.3%. These comparisons can be of aid to assess and to rule out postoperative complication comparison between two procedures under this study.

## 4. Conclusion and Clinical Significance

Based on the evidence generated in this study, TPRK presents a dominance over the conventional PRK in the accuracy of the procedure as inferred from the predictability outcome comparison measures. The clear lead of TPRK over the conventional PRK in all the secondary outcomes is a sign of less postoperative complications with the TPRK procedure. A greater acceptance of the patients in terms of pain levels and postoperative satisfaction levels of the TPRK is very promising and raises confidence in them for repeating the procedure. In conclusion, TPRK can be suggested as an alternative surgical procedure to the conventional PRK for all types of refractive errors, in terms of its accuracy, postoperative complications, patient comfort, and acceptance. Industrial funding was an important aspect to investigate when it comes to a technology-based intervention. Research reports can be biased with industrial influence with an aim of commercial gain from it. An author of an included study [32]. in this review had its author working for the laser technology being used and the author reported no clinical benefit of TPRK over PRK, except for better intraoperative experience, to the patient.

## 5. Limitations

In this study, only 2 studies [29, 38]. were a randomized, double-blinded trial and all other 11 studies [11, 29–37]. were nonrandomized studies. This unveils a serious lack of randomized clinical trials identifying clinical outcomes of both the procedures compared in this study. The outcome measurements from the studies were of different types and the follow-up periods differed greatly among the included articles which had influenced the reporting in this analysis study. Therefore, the use of indicators with good representativeness and sensitivity using identical comparative methods is recommended for future clinical evidence-based combined analysis.

Due to difference in the study protocols, regional back grounds, methodological heterogeneity among studies can be seen as the outcome comparisons. Clinical heterogeneity, for instance, arises from the postoperative visual acuity (VA) measure which influences the postoperative measures of VA and different sample sizes. Studies with large samples with unified reporting formats are advisable to generate reliable evidence. As the technology is a very dynamic medium, the laser technique used in the included studies is not of the most recent generation laser units. This is a factor influencing the clinical outcome of the surgical time comparison studies in which the different laser generation units are used. Furthermore, as only few randomized double blinded RCTs are published, resulting in safety outcomes to be underpowered, if only RCTs had been analyzed. This meta-analysis had added different types of RCTs to outweigh the disadvantages of including only randomized RCTs. The additional inclusion of the observational studies might outweigh the disadvantages of including only RCTs [39]. Studies covering concerning patients' satisfaction were limited. Furthermore, future analysis of the heterogeneous manner of clinical study outcomes reporting is profitable in generating explicit clinical evidence. More studies should be initiated with the younger generation of laser techniques to report on the laser energy load, surgical time, and the cost of the procedure.

## Figures and Tables

**Table 1:** The summary of the attributes included studies.

#	Study	Methodology/study type	Follow-up (months)	Treated eyes		Total treated eyes	Industrial funding	Laser used	Remarks
				T P R K	P R K				
1	Ghobashy et al. [29]	Prospective case-control	6	24	24	48	Not reported	Schwind Amaris 500 E excimer laser.	A faster early postoperative visual recovery is suggested in this study. It is reported in this study that TPRK can be a more safe, less painful, and an effective alternative to conventional PRK

2	Naderi et al. 2016 [30]	Prospective case-control	6	170	170	340	Not reported	Schwind Amaris 500 E excimer laser	Reports the superiority of TPRK in comparison to conventional PRK, in terms of significant safety and efficacy indices

3	Kaluzny et al. [31]	Prospective case-control	3	173	103	276	Not reported	Amaris excimer laser, version 750 S	The similarity in results between TPRK and PRK within 3 months of postoperative follow-up in the refractive visual outcomes are concluded in this study

4	Antonios et al. [32]	Retrospective comparative	12	59	59	118	Author employee of Schwind eye tech	Schwind Amaris excimer laser	Visual, refractive, and safety comparison results of this study within 3 months of the surgery, reports similar refractive results with both the procedures

5	Bakhsh [33]	Prospective case-control comparative	6	100	100	200	Not reported	Schwind Amaris excimer laser 750 S	Though this study reports the significant all primary and secondary outcomes in the early postoperative period, the study concludes that TPRK and PRK give similar results after a 6-month period

6	Özülken and Ilhan [11]	Retrospective comparative	12	54	54	108	Not reported	Amaris excimer laser version 750 S	Similar results between both TPRK and PRK in terms of postoperative CDVA, SE, asphericity, and higher order abbreations is stated by this study

7	Gadde [34]	Retrospective case-control study	3.5	67	48	115	Not reported	Amaris excimer 500 E laser	On comparing the postoperative UDVA, BCVA, safety, safety index, efficacy, and efficacy index, this study reports that the two procedures have no superiority over each other in terms of long-term results

8	Gaeckle, [35]	Prospective clinical observational	1.5	100	100	200	Not reported	WaveLight® EX500 excimer laser	This study emphasizes that both procedures appear to be safe and effective methods but suggests that TPRK offered faster visual recovery and epithelial healing and was associated with less pain compared to PRK

9	Rodriguez [36]	Prospective cohort study	40	16	16	32	Not reported	Schwind Amaris 750 S excimer laser	The authors here highlight that there is no statistically significant differences in any visual and refractive results but reports faster healing and recovery in TPRK

10	Ellakava [37]	Prospective comparative	3	50	50	100	Not reported	Amaris excimer laser	A better visual outcome, faster healing time, and less postoperative haze with TPRK over conventional PRK is reported in this study

11	Gharieb, [38]	Prospective double blinded	12	27	27	54	Not reported	Schwind Amaris 1050 Hz	PRK and TPRK are stated in this study to having comparable results regarding safety and efficacy

12	Nasseri [29]	Clinical trial double blinded	3	60	60	120	Not reported	Schwind Amaris 750 S excimer laser	The superiority of the TPRK method over the PRK within 3 months of follow-up period in terms of UDVA, BCVA, and SE is reported in this study

## Data Availability

Data used in this study are extracted from the included studies and is available on request.

## Additional Points

*What Was Known.* TPRK and PRK have not been a subject of a meta-analysis to provide clear and comprehensive clinical evidence of procedural preference with the main and secondary outcomes in concern. Most of the studies show significantly better results in early postoperative outcomes with TPRK and later become comparable to the PRK in the efficacy and safety measures. *What this paper adds*. An updated clinical evidence based on this meta-analysis which asserts that TPRK is more accurate than PRK as revealed from the predictability comparisons. TPRK and PRK are comparable in the efficacy and safety in the late follow-up periods. All the secondary outcome comparisons establish the clear supremacy and acceptance of TPRK.
